# THE RELATIONSHIP BETWEEN RETIREMENT AND CARDIOVASCULAR DISEASE: A MEDIATION ANALYSIS IN THE U.S. AND SOUTH KOREA

**DOI:** 10.1093/geroni/igae098.4279

**Published:** 2024-12-31

**Authors:** Gayoung Choi, Ruijia Chen, Hee Man Park, Harold Lee

**Affiliations:** Sungkyunkwan University, Seoul, Republic of Korea; Boston University, Lexington, Massachusetts, United States; Pennsylvania State University, State College, Pennsylvania, United States; Penn State, University Park, Pennsylvania, United States

## Abstract

Retirement has been linked to increased risk for cardiovascular disease (CVD), but little is known about the pathways underlying the association. Moreover, most studies have been conducted in Western countries, and their findings might be less generalizable to Eastern countries due to different social contexts and labor and employment environment. We examined 1) whether retirement status is associated with a higher risk of CVD in South Korea and the U.S., and 2) whether health (e.g., hypertension, diabetes, and obesity), behavioral (e.g., physical activity, alcohol consumption, and smoking), and psychological factors (e.g., loneliness, depressive symptoms, and quality of life) mediate these associations. Data were drawn from the Health and Retirement Study (HRS, 2002–2014; n=11,855) and the Korean Longitudinal Study of Aging (KLoSA, 2006–2018; n=4,908). We analyzed the data using cox proportional hazard model, controlling for socio-demographic factors, health conditions, health behavior, and social isolation. We also performed causal mediation analyses based on counterfactual framework. After adjusting for all covariates, retirement (vs. employment) was associated with an increased likelihood of developing CVD (Hazard Ratio (HR): 1.22, 95% CI: 1.00-1.49) in Korea but not in the U.S. Regarding potential mediators, several physical health factors accounted for a proportion of the association in KLoSA: hypertension (17.7%, p< 0.01), diabetes (12.1%, p< 0.05), and obesity (3.7%, p=0.01). The psychological pathway, specifically quality of life, also mediated the association (4.8%, p< 0.05). Our findings highlight that public health interventions targeting physical and mental health could be an important strategy for preventing CVD among retirees, especially in Korea.

